# Relationship between Urinary Parameters and Double-J Stent Encrustation

**DOI:** 10.3390/jcm12155149

**Published:** 2023-08-06

**Authors:** Jose Luis Bauzá, Paula Calvó, Francesca Julià, Jorge Guimerà, Ana Isabel Martínez, Antonio Tienza, Antonia Costa-Bauzá, Pilar Sanchís, Félix Grases, Enrique Pieras

**Affiliations:** 1 Urology Department, University Hospital Son Espases, 07120 Palma de Mallorca, Spainanai.martinezm@ssib.es (A.I.M.); antonio.tienza@ssib.es (A.T.);; 2 Nefro-Urologic Diseases Research Group, Fundación Instituto de Investigación Sanitaria Islas Baleares (IdISBa), 07120 Palma de Mallorca, Spain; 3 Laboratory of Renal Lithiasis Research, University Institute of Health Sciences Research (IUNICS-IdISBa), University of Balearic Islands, 07122 Palma de Mallorca, Spainf.julia@uib.es (F.J.);

**Keywords:** urinary stent, encrustation, indwelling time

## Abstract

(1) Background: This study aimed to determine the relationship between metabolic urine conditions and the formation, severity, and composition of encrustations in ureteral stents. (2) Methods: Ninety stone-former patients requiring a double-J stent were prospectively enrolled. We collected 24 h metabolic urine samples and demographic data, including indwelling time and previous stone composition. The total deposit weight was obtained, and a macroscopic classification according to the degree of encrustation (null, low, moderate, and high) was created, allowing for intergroup comparisons. Stereoscopic and scanning electron microscopy were performed to identify the type of embedded deposits (calcium oxalate, uric acid, and infectious and non-infectious phosphates). (3) Results: In total, 70% of stents were encrusted; thereof, 42% had a moderate degree of encrustation. The most common encrustation type was calcium oxalate, but infectious phosphates were predominant in the high-encrustation group (*p* < 0.05). A direct correlation was observed between the purpose-built macroscopic classification and the encrustation weights (*p* < 0.001). Greater calciuria, uricosuria, indwelling time, and decreased diuresis were observed in stents with a higher degree of encrustation (*p* < 0.05). The urinary pH values were lower in patients with uric acid encrustations and higher in those with infectious phosphate encrustations (*p* < 0.05). When compared to non-encrusted stents, patients with calcium-oxalate-encrusted stent showed greater calciuria, phosphaturia, indwelling time, and reduced diuresis; patients with uric-acid-encrusted stent showed greater uricosuria; and patients with infectious and non-infectious phosphate encrustation showed greater urinary pH (*p* < 0.05). (4) Conclusions: Metabolic urine conditions play a critical role in the formation, composition, and severity of double-J stent encrustation.

## 1. Introduction

Since being first described by Finney in 1978, the double-J ureteral stent has become one of the most commonly used tools by urologists because of its wide range of applications [1]. The most common indication for ureteral stents is the drainage of an obstructed upper urinary tract, although it is also commonly used to dilate the ureters to aid instrumentation and to prevent occlusion following endourological procedures or even to provide a scaffold for healing after reconstructive surgery [2,3,4].

Extensive studies have led to the use of novel designs and materials; however, the utilization of double-J stents may still have some drawbacks, as up to 80% of patients experience complications following placement [5,6]. Fortunately, most of these complications are mild, such as discomfort or hematuria, but more severe side effects may occur, such as urinary tract infection, encrustation, chronic obstruction, and consequent loss of the renal unit [7,8]. These issues not only impact the patient’s quality of life but can also exert a significant economic toll, as additional procedures may be required to treat and remove encrusted stents [9,10].

Encrustation involves the deposition of crystals on the interior and exterior surfaces of the ureteral stent [11]. This could lead to severe complications, especially when a prolonged indwelling time or forgotten stent occurs, as in up to 13% of cases [12]. The presence of severe encrustation could jeopardize the stent’s tensile strength, facilitating its rupture during removal, and could also induce ureteral damage or avulsion, urinary tract infection, or even the loss of the renal unit when the crystal depositions chronically obstruct stent drainage [11,13,14].

The mechanisms involved in the development of encrustations are not yet well known, although it is suggested to be a highly complex multifactorial process. Biofilm formation was supposed to play a critical role, as biofilms may be composed of urease-producing bacteria (Proteus, Pseudomonas, Klebsiella, etc.) with the ability to split urea into ammonium, thus raising the urinary pH and allowing the precipitation of struvite on the double-J stent surface [15]. However, encrustations may also occur in sterile conditions, in fact, the most common composition identified on double-J stents is calcium oxalate [16,17]. Therefore, urine conditions, such as pH, supersaturation of crystallizing substances, and deficit of crystallizing inhibitors, may play a major role [16,17].

These previously mentioned mechanisms have not been directly studied as most of the encrustation formation theories have been derived from the urinary stone formation theories. Considering that previous studies showed a high concordance between stone’s and stent encrustation’s compositions, as well as similar changes for double-J stent encrustations and stone compositions according to different age and gender distributions [17,18], the assumption that the same lithogenic factors act similarly in stone formation and double-J stent encrustation could be made.

The relationship between specific metabolic urine conditions and the formation of different stone types was previously established [19]. Consequently, specific prevention treatments were proposed, thus reducing urinary stones’ recurrence [4]. Knowing the metabolic urinary alterations related to double-J stent encrustation could lead to individualized preventive treatments, which could reduce related complications. Nonetheless, this topic has not been studied in recent years and remains to be confirmed. We aimed to determine the relationship between metabolic urine conditions and the formation, severity, and composition of encrustations on double-J stents in stone-former patients.

## 2. Materials and Methods

This study was conducted between January 2018 and December 2021 and was based on a unicenter prospective controlled model. Data on 90 consecutive stone-former patients requiring double-J placement, irrespective of their emergent or programmed fashion, were gathered. Only patients more than 18 years old and without previous treatment with any urolithiasis-specific drug were eligible. Those patients were followed until double-J stent removal. The patient’s demographic features (age; sex; comorbidities such as arterial hypertension, dyslipidemia, and diabetes; urine culture; stone episode; indwelling time; and previous stone composition, when available) were registered, as well as the need for ancillary procedures for double-J stent removal. Urine samples for urine culture were collected at the time of presentation before any antibiotics or other treatments were administered. Once collected, the samples were cold-stored until direct examination, and a posterior culture and an antibiogram were performed.

All patients were required to collect a 24 h metabolic urine sample while the double-J stent was in place, specifically between 2 and 3 weeks after insertion. The studied parameters were pH, diuresis, urine concentration, and urine total amounts of creatinine, urate, calcium, phosphate, magnesium, oxalate, and citrate. The 24 h urine collection was carried out in a thymol-based sterile container and immediately stored at −20 °C to preserve the sample. Diuresis was recorded for 24 h, although the urine collected during the first 2 h was used for pH determination with a glass-based-electrode pH meter (Crison, Hach Lange^®^, Barcelona, Spain), to avoid the occurrence of pH changes due to the precipitation of calcium salts, which may happen during the first 24 h of storage. Oxalate and citrate levels were measured using the R-Biopharm^®^ enzymatic test with the kits 10755699035 and 10139076035, respectively. Phosphorus, calcium, and magnesium levels were determined using atomic emission spectroscopy in a spectrophotometer. Creatinine and uric acid values were determined using the Roche^®^ modular assay with the reagents 11875663216 and 11875426216, respectively.

**Figure 1:**
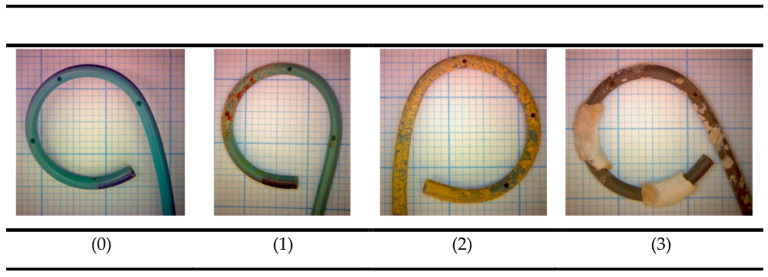
Macroscopic classification according to encrustation grade: 0, no deposit; 1, low degree of encrustation (<25% of the stent); 2, moderate degree of encrustation (<50% of the stent); 3, high degree of encrustation or deposit block (>50% of the stent).

The Angiomed double-J stent, UROSOFT ureteral stent set (REF 57410030), was used for all patients in this study. The mean indwelling time of the double-J stents included in this study was 89 days. After removal, the stent was divided into two identical parts (proximal and distal) and studied following the protocol previously published by our group [20]. In the first place, double-J stents were photographed with a stereoscopic microscope. Subsequently, a semi-quantitative classification was created, and three independent laboratory technicians classified the stents into four categories according to the degree of encrustation observed (from 0 to 3, with 0 being the absence of deposits, 1 being a low degree of encrustation (up to 25% of the stent), 2 being a moderate degree of encrustation (up to 50% of the stent), and 3 being a high degree of encrustation or deposit block (more than 50% of the stent), as shown in Figure 1). Concordance between all of the technicians’ classifications was very high, but whenever a discrepancy existed, the stent was classified in the most voted category. After that, each stent was weighed, and the total deposit weight was obtained by subtraction of the weight of an unused catheter. Later, stereoscopic and scanning electron microscopy were performed to identify the type of embedded deposits (calcium oxalate, uric acid, infectious phosphates, and non-infectious phosphates). Finally, the stents were divided into multiple small fragments to facilitate the dissolution of the deposits with 2 M hydrochloric acid (HCl) (except for those with uric acid and/or urate encrustations) to determine the amounts of calcium, phosphorus, and magnesium present in the encrustations using inductively coupled plasma–atomic emission spectrometers (ICP-AESs).

Comparisons were made to identify the correlation between encrustation’s grade and type with the demographic features. The prevalences of each encrustation type were studied, as well as their distribution across a severity scale. Finally, urine metabolic conditions were compared between encrusted and non-encrusted stents, none–low and moderate–high encrusted stents, and for every encrustation type.

### 2.1. Statistical Analysis

Normality plots and graphs were used to assess the data distribution. Continuous variables are represented as median and interquartile range (IQR), and categorical variables are expressed as absolute numbers and percentages. Statistical analyses were performed using SPSS 25.0 (SPSS Inc., Chicago, IL, USA). Intergroup comparisons were performed using a Mann–Whitney U test. A chi-square or Fisher’s exact test was used to compare categorical variables. The correlations between variables were assessed with the Spearman test. A two-tailed *p*-value of <0.05 was considered statistically significant.

### 2.2. Data Availability

The data associated with this paper are not publicly available but are available from the corresponding author on reasonable request.

## 3. Results

Ninety stone patients requiring a double-J insertion were included in this study; most of them were men, with a median age of 55 years old. The majority of the patients were recurrent stone formers, with at least a previous double-j stent insertion. The median indwelling time in our study was 58 days. The demographic features are summarized in Table 1.

**Figure 2:**
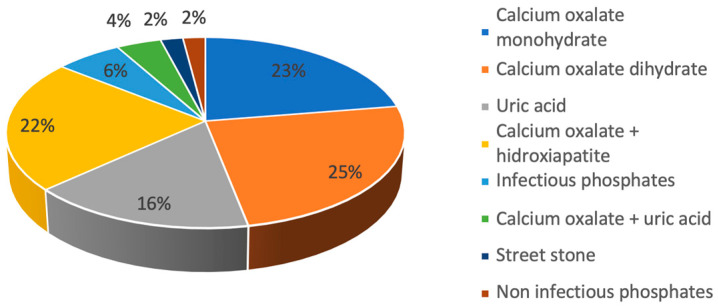
Composition of previous stones.

No relationship was found between the presence of any comorbidity (hypertension, dyslipidemia, type II diabetes) and the severity of encrustation (*p* > 0.05). Recurrent lithiasic patients showed greater encrustation when compared with first-episode patients (0.06 g [0.01–0.15] vs. 0.02 g [0–0.06]; *p* = 0.047). Twenty (22.2%) patients had a positive urine culture at the time of double-J stent placement. There was no relationship between a positive urine culture and the presence of infectious phosphate encrustation (*p* = 0.2), or with other types (*p* = 0.9) or grades of encrustation (*p* = 0.8). Forty-nine (54.4%) patients had a previous stone composition analysis, with calcium oxalate begin the most common composition (Figure 2). The concordance between the stone’s and the encrustation’s composition was very high, as the compositions were similar in 85.7% of the cases (*p* = 0.01).

**Figure 3:**
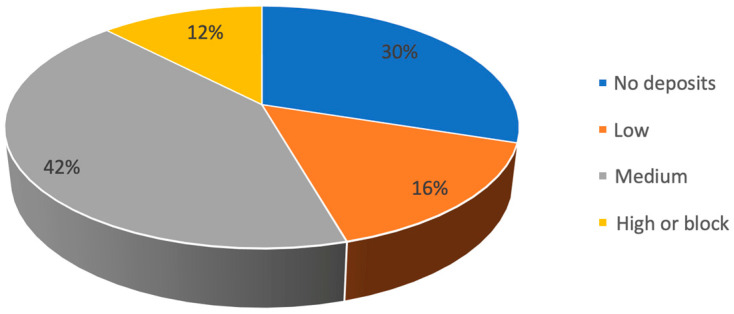
Encrustation grade frequencies.

**Figure 4:**
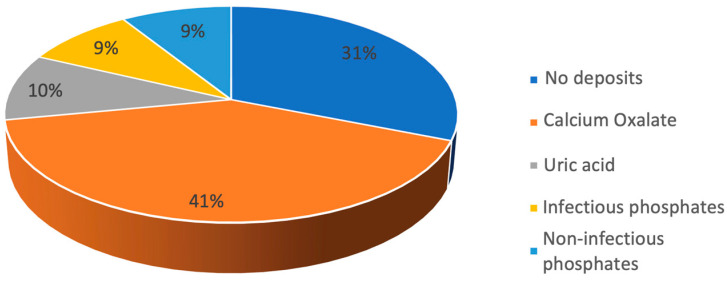
Encrustation type frequencies.

**Figure 5:**
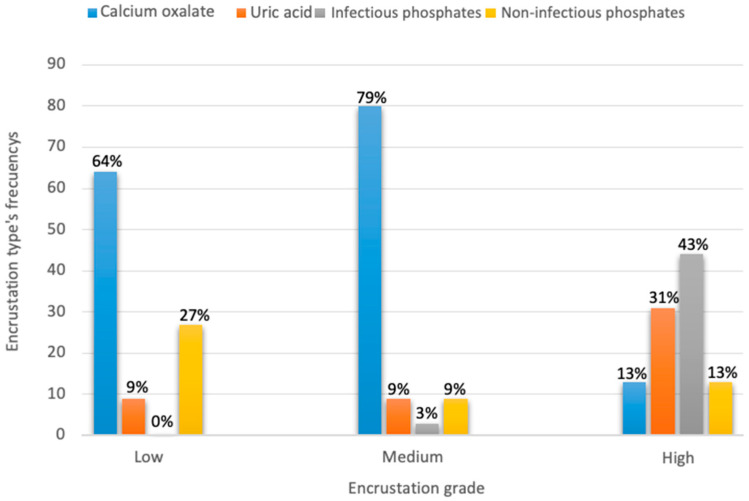
Encrustation type frequencies classified according to the degree of encrustation.

Sixty-three (70%) of the stents presented some degree of encrustation; most (42%) had a moderate grade of encrustation (Figure 3). In general, the most common encrustation type found was calcium oxalate (CaOx) (Figure 4). CaOx was also the most common type in the low- and medium-encrustation grades (63.3% [n = 9] and 80% [n = 31]; *p* < 0.05), while infectious phosphates were more common in the high-encrustation grade (43.8% [n = 5]; *p* < 0.05) (Figure 5).

**Figure 6:**
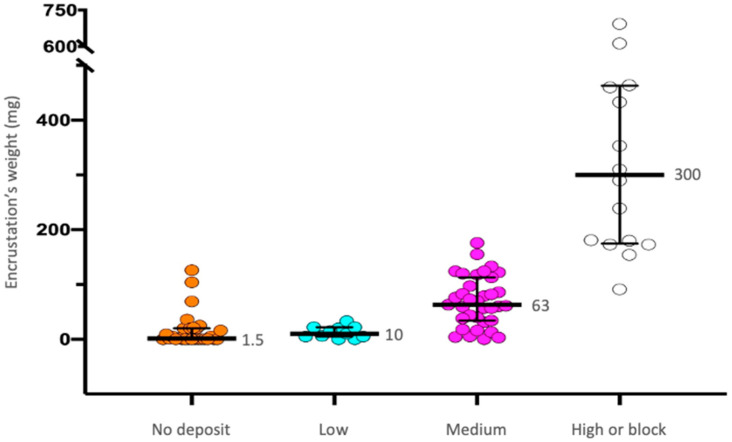
Positive association between our purpose-built macroscopic classification and the weight of the encrustation.

A direct association was observed when comparing our purpose-built macroscopic classification and the encrustation weights (rs = 0.777; *p* < 0.001) (Figure 6). Accordingly, higher amounts of calcium, phosphorus, and magnesium were noticed with increasing double-J stent encrustation grade for each type of encrustation (*p* < 0.001) (Table 2). In seven cases (7.7%), conventional cystoscopy alone was not sufficient for double-J stent removal, requiring mechanical or laser lithotripsy of the distal end blocks. All these cases were classified as having a high-encrustation grade, and the major encrustation type was infectious phosphates (4/7).

In order to elucidate the urine conditions related to encrustation formation, we divided the patients into two groups (non-encrusted vs. encrusted). Patients with encrusted double-J stents showed significantly less diuresis, higher phosphaturia, oxaluria, and longer indwelling times (*p* < 0.05) (Table 3).

Next, in order to identify those alterations in the urine conditions that related to a higher degree of double-J stent encrustation, we classified the patients into two groups (none-to-low vs. moderate-to-high stent encrustation grade) and compared the 24 h urinary biochemistry and indwelling times, observing greater calciuria, uricosuria, phosphaturia, citraturia, and oxaluria, as well as decreased diuresis in those stents with a higher degree of encrustation (*p* < 0.05). Although not statistically significant, a tendency for greater indwelling times was observed in highly encrusted double-J stents (*p* = 0.086) (Table 4).

Subsequently, to explore the urine conditions involved in the formation of each encrustation type, we performed two investigations. First, we analyzed the differences in the 24 h urinary biochemistry according to the composition of the encrustation and observed lower urinary pH values in patients with uric acid encrustations and higher pH values in patients with infectious phosphate encrustations (*p* < 0.05). Significant differences were also identified when comparing indwelling times as patients with uric acid and infectious phosphates showed significantly lower indwelling times (*p* < 0.05) (Table 5).

Finally, we evaluated the differences between each encrustation type’s 24 h urinary biochemistry and that of non-encrusted stents. Patients with calcium oxalate encrustation showed greater calciuria, phosphaturia, and citraturia, as well as an increased indwelling time and reduced diuresis, than patients without encrustations on their double-J stents (*p* < 0.05) (Table 6). Patients with uric acid encrustations showed greater uricosuria and reduced diuresis (*p* < 0.05). The indwelling time was slightly reduced in patients with uric acid encrustations. Although these differences were statistically significant, they may not be clinically significant. The pH was also slightly reduced in patients with uric acid encrustations when compared to non-encrusted patients, but it was not statistically significant (*p* > 0.05) (Table 7). Patients with infectious phosphate encrustations showed a greater pH than non-encrusted patients (*p* < 0.05). Although not significant, a tendency was set for lower diuresis, lower uricosuria, lower citraturia, and lower indwelling times (*p* > 0.05) (Table 8). Finally, patients with non-infectious phosphate encrustations showed a greater urinary pH when compared to non-encrusted double-J stent patients (*p* < 0.05); also, a trend for lower uricosuria was observed in patients with non-infectious phosphate encrustations (*p* > 0.05) (Table 9).

## 4. Discussion

In our results, 70% of stents were encrusted; 42% had a moderate degree of encrustation. The most common encrustation type was calcium oxalate, but infectious phosphates were predominant in the high-encrustation group. A direct association was observed between the purpose-built macroscopic classification and the encrustation weights. Greater calciuria, uricosuria, indwelling time, and decreased diuresis were observed in stents with a higher degree of encrustation, and urinary pH values were lower in patients with uric acid encrustations and higher in those with infectious phosphate encrustations. When compared to non-encrusted stents, patients with calcium-oxalate-encrusted stent showed greater calciuria, indwelling time, and reduced diuresis; patients with uric acid encrustation showed greater uricosuria, and patients with infectious and non-infectious phosphate encrustation showed greater urinary pH. Metabolic urinary conditions could play an important role in the multifactorial process of double-J stent encrustation in patients with urolithiasis.

Double-J stents are extremely versatile tools that exhibit a wide range of indications. Their efficacy in allowing drainage of the obstructed upper urinary tract, either due to lithiasis or other causes, such as malignant and non-malignant stenosis, has been widely proven [2,3,4,21]. Stents have also been useful as a healing scaffold following reconstructive surgery [22]. In addition, the constant and frenetic evolution of new endourological techniques for the treatment of kidney stones and other diseases has expanded their indications further, thus increasing the number of double-J stents used worldwide [23]. Despite its great number of applications in several diseases, it is in urolithiasis that double-J stents are the most important. The current indications include acute drainage of obstructing urinary stones (which is mandatory whenever a bilateral obstruction is present, a solitary kidney, uncontrollable pain, or when a urinary tract infection is associated), stent insertion after a ureteroscopy (which can be omitted in selected cases of uncomplicated procedures), and prophylactic stenting prior to procedures like external shock-wave lithotripsy or flexible ureterorenoscopy (which can prevent steinstrasse occurrence and improve stone-free rates) [2,4].

However, double-J stent use also has some complications. Although the majority of side effects are mild, potentially serious complications, such as urinary tract infection or encrustation, are common [5,11]. Encrustation is the growth of crystals on the surface of the stent, which can lead to chronic obstruction and the consequent massive function loss of the obstructed renal unit or even death if left untreated [7,8]. These issues could impact the patient’s quality of life besides exerting a significant economic toll [9] since additional procedures may be required to treat and remove highly encrusted stents, as has been recently published [10].

Encrustation requires a complex multifactorial mechanism, which is not yet well known. Biofilm formation and colonization by urease-producing bacteria (Proteus, Pseudomonas, Klebsiella, etc.) play a major role in encrustation formation [11,24]. Nevertheless, encrustations have also been observed under sterile conditions; in fact, the most common composition identified on double-J stents is not struvite but calcium oxalate, suggesting that other contributing factors are involved [16,25]. Probably, urine conditions, such as pH, supersaturation of crystallizing substances, and deficit of crystallizing inhibitors, play a major role, as double-J stents remain constantly in contact with the urine [16,17]. However, this topic has never been directly investigated until the present study. Many other factors have been linked to encrustation formation, with indwelling time being the most studied [2,11]. El-Faqih et al. were the first to observe how the encrustations grew over indwelling time, with 76.3% of the stents encrusted when removed after 12 weeks [26]. Our results were similar, as 70% of the stents in our cohort were encrusted after a median indwelling time of almost 60 days. Subsequently, other authors, such as Kawahara et al. and Legrand et al., found comparable results in their studies [27,28]. Other investigated risk factors for double-J stent encrustation are recurrent stone formers and conditions related to urolithiasis (such as malabsorptive disorders, diet, etc.); recurrent urinary tract infections, diabetes mellitus, and chronic kidney disease (as these may increase the urinary bacterial load); and pregnancy [11]. In our results, we observed grater encrustation when comparing recurrent lithiasic patients with first-episode patients, in concordance with previously published data. On the other hand, the presence of type II diabetes did not impact the severity or the type of encrustation in our study, which could be explained by the low number of type II diabetic patients included in this study.

Bouzidi et al., in their prospective study of 658 ureteral stents, observed the effect of indwelling time on the encrustation composition. According to their results, calcium oxalate was predominant during the first 30 days of indwelling, while uric acid and infectious phosphates increased gradually over time [17]. Conversely, in our study, uric acid and infectious phosphates were found in stents with shorter indwelling times. Interestingly, in our cases, uric acid and infectious phosphates were also the most predominant, with a high degree of encrustation. Thus, in our opinion, attention should be given to these patients as their complication risk is higher, and methods to prevent encrustation or treat them faster should be employed.

As previously mentioned, Bithelis et al. observed that calcium oxalate was the most common encrustation composition [25]. Later, Roupret et al. and Sighinolfi et al. also found similar results [18,29]. Comparable results were also found in the present study, with calcium oxalate being the most common encrustation type (41%). Bouzidi et al. also observed calcium oxalate as the most common encrustation type; moreover, they observed that the variations in encrustation compositions that occurred according to variations in age and gender were very similar to those occurring in the kidney stones themselves, suggesting that the same mechanisms involved in stone formation are involved in encrustation formation [17]. However, only indirect data exist to support this theory, as it had never been directly investigated until our study, which showed that the metabolic urinary alterations found for a given encrustation composition were very similar to those that could be expected for the same stone composition, supporting the theory of Bouzidi et al.

Roupret et al. also showed a high concordance between encrustation and stone compositions (78%) [18]. In the present study, the concordance was even higher, reaching almost 86%, again supporting the theory that the lithogenic factors involved in stone and encrustation formation could be the same. Furthermore, the results of our study suggest a new role for 24 h urine metabolic analysis, as it could be very useful in identifying patients at a high risk of encrustation, especially when a prolonged indwelling time is expected, allowing for targeted and personalized treatment, thus reducing subsequent complications and improving patients’ quality of life.

Finally, in a recent double-blind, multicenter, placebo-controlled trial, Torrecilla et al. showed promising results using a novel compound (composed of a urine acidifier and a crystallization inhibitor) to reduce ureteral stent encrustation [30]. This shows that, by modulating urine conditions, ureteral stent encrustations can be prevented, supporting our theory of encrustation formation.

This study had some limitations. The need for multiple subclassifications resulted in a few underpopulated groups, which could have had some impact on our results. Thus, a multicenter study with a larger population could provide more robust results. In addition, no prophylactic measures to reduce double-J stent encrustation were investigated, which should form the basis of future studies. Also, the impact of treatments for other pathologies like hypertension or diabetes in 24 h urine analysis was not studied.

In the present study, we confirmed previous known risk factors for double-J stent encrustation, such as prolonged indwelling time and recurrent urolithiasis. Moreover, we are the first, to the best of our knowledge, to directly investigate the role of metabolic urinary conditions in the formation, severity, and type of encrustation present on double-J stents. Our results point out the importance of 24 h urine biochemistry in identifying high-encrustation-risk patients on whom an extensive effort has to be made to prevent encrustation, either by reducing indwelling time until definitive treatment or by using prophylactic measures, such as phytate, a calcium oxalate lithiasis inhibitor [31], or a urine acidifier to prevent non-infectious phosphate lithiasis [4], which are, according to our findings, one of the fastest encrustations to grow and the most commonly encountered encrustation type in the high degree of encrustation group.

## 5. Conclusions

Metabolic urine conditions play a critical role in the formation, composition, and severity of double-J stent encrustation. Reduced diuresis and increased phosphaturia, oxaluria, and indwelling time may play a critical role in encrustation formation. For the degree of encrustation, reduced diuresis and increased calciuria, oxaluria, uricosuria, phosphaturia, and indwelling time, may play an additional major role. Greater pH could promote infectious and non-infectious phosphate encrustations. Lower indwelling times were found for uric acid and infectious phosphate encrustations.

## Figures and Tables

**Table 1:** Baseline characteristics of patients.

Gender	Male	57.3% (43)
	Female	42.7% (32)
Age	55 (28–84)	
Comorbidities	Hypertension	37.3% (28)
	Type II diabetes	10.6% (8)
	Dyslipidemia	21.3% (16)
Stent placement reason	Emergent	65.6% (59)
	Programmed	34.4% (31)
Stone episode	First	37.7% (34)
	Recurrent	62.2% (56)
Indwelling time (days)	58 (26–102)	
Urine culture	Negative	77.7% (70)
	Positive	22.2% (20)

**Table 2:** Calcium, phosphorus, and magnesium amounts according to each type and grade of encrustation.

Encrustation Type	Encrustation Grade								
	Calcium (µmol) Median			Phosphorus (µmol) Median			Magnesium (µmol) Median		
	Low	Medium	High	Low	Medium	High	Low	Medium	High
Calcium oxalate	46.4	351	876	3.74	12.8	30.3	0.23	0.87	1.27
Infectious phosphates	-	486	1687	-	784	1202	-	366	235
Non-infectious phosphates	4.99	390	3607	8.1	298	2674	0.43	10.4	112

**Table 3:** Urinary biochemistry (24 h) and indwelling time comparison between encrusted and non-encrusted stents.

	Non-Encrusted (n = 25)	Encrusted (n = 65)	
Urinary Parameters	Median (IQR)	Median (IQR)	*p*
pH	5.6 (5.2–6.5)	5.8 (5.5–6.7)	0.255
24 h diuresis (mL)	2000 (1450–2700)	1800 (1225–2200)	0.041
Urine creatinine (mg/dL)	57 (38–99)	80 (48–99)	0.178
Urine creatinine (mg/24 h)	1227 (782–1490)	1199 (917–1708)	0.377
Urine urate (mg/dL)	28 (13–43)	34 (21–48)	0.122
Urine urate (mg/24 h)	534 (376–730)	551 (362–758)	0.869
Urine calcium (mg/dL)	6.9 (3.9–10.5)	8.3 (6.2–13.1)	0.111
Urine calcium (mg/24 h)	132 (89–234)	165 (100–227)	0.472
Urine phosphate (mg/dL)	-	43 (30–68)	0.054
Urine phosphate (mg/24 h)	669 (409–928)	772 (594–945)	0.283
Urine magnesium (mg/dL)	3.4 (2.0–6.2)	4.3 (2.2–6.1)	0.682
Urine magnesium (mg/24 h)	86 (56–117)	85 (69–109)	0.638
Urine oxalate (mg/L)	13.8 (9.6–19.8)	18.0 (14.1–23.9)	0.025
Urine oxalate (mg/24 h)	28 (16–39)	26 (21–41)	0.779
Urine citrate (mg/L)	153 (99–278)	232 (117–409)	0.154
Urine citrate (mg/24 h)	270 (221–608)	476 (257–758)	0.115
Indwelling time (days)	35 (16–92)	67 (37–103)	0.046

**Table 4:** Urinary biochemistry (24 h) and indwelling time comparison between low-grade encrusted and high-grade encrusted stents.

	Encrustation Grade		
	None–Low (n = 41)	Medium–High (n = 49)	
Urinary Parameters	Median (IQR)	Median (IQR)	*p*
pH	6.0 (5.3–6.7)	5.7 (5.4–6.5)	0.633
24 h diuresis (mL)	2100 (1500–2525)	1700 (1100–2200)	0.029
Urine creatinine (mg/dL)	54 (37–90)	83 (59–106)	0.006
Urine creatinine (mg/24 h)	1106 (794–1484)	1349 (990–1712)	0.044
Urine urate (mg/dL)	24 (14–43)	37 (22–49)	0.015
Urine urate (mg/24 h)	500 (345–720)	574 (396–778)	0.262
Urine calcium (mg/dL)	7.1 (3.9–8.8)	9.0 (6.6–16.7)	0.018
Urine calcium (mg/24 h)	105 (82–214)	176 (105–244)	0.140
Urine phosphate (mg/dL)	35 (24–47)	47 (30–70)	0.021
Urine phosphate (mg/24 h)	701 (481–919)	789 (576–951)	0.268
Urine magnesium (mg/dL)	3.4 (2.1–5.2)	4.5 (1.6–6.9)	0.133
Urine magnesium (mg/24 h)	77 (57–105)	93 (70–119)	0.077
Urine oxalate (mg/L)	14 (10–20)	19 (15–23)	0.041
Urine oxalate (mg/24 h)	28 (21–41)	26 (20–42)	0.913
Urine citrate (mg/L)	158 (100–314)	267 (151–446)	0.049
Urine citrate (mg/24 h)	270 (226–590)	495 (320–859)	0.049
Indwelling time (days)	50 (16–93)	66 (35–116)	0.086

**Table 5:** Urinary biochemistry (24 h) and indwelling time comparison according to encrustation composition.

	Calcium Oxalate (n = 38)	Uric Acid (n = 10)	Infectious Phosphates (n = 5)	Non-Infectious Phosphates (n = 12)	
Urinary Parameters	Median (IQR)	Median (IQR)	Median (IQR)	Median (IQR)	*p*
pH	6.0 (5.7–6.7)	5.3 (5.0–6.4)	6.6 (6.2–6.8)	5.9 (5.5–7.2)	0.010
24 h diuresis (mL)	1800 (1400–2200)	1600 (1000–2600)	1533 (1200–1830)	2050 (1525–2650)	0.382
Urine creatinine (mg/dL)	82 (42–99)	89 (52–111)	67 (39–90)	58 (39.7–63.7)	0.581
Urine creatinine (mg/24 h)	1067 (845–1712)	1323 (829–1510)	1045 (847–1623)	1176 (919–1472)	0.783
Urine urate (mg/dL)	34 (18–40)	50 (29–420)	28 (17–34)	23(17–31)	0.081
Urine urate (mg/24 h)	543 (339–749)	733 (529–3434)	417 (298–577)	448 (414–692)	0.019
Urine calcium (mg/dL)	8.1 (6.6–13.0)	6.4 (3.6–12.6)	10.5 (6.8–13.7)	8.5 (7.2–11.1)	0.313
Urine calcium (mg/24 h)	187 (80–273)	142 (77–212)	167 (78–253)	227 (131–277)	0.298
Urine phosphate (mg/dL)	43 (34–68)	50 (32–75)	36 (28–62)	30 (26–39)	0.651
Urine phosphate (mg/24 h)	725 (648–937)	855 (657–1133)	554 (345–737)	773 (488–893)	0.367
Urine magnesium (mg/dL)	3.5 (2.2–6.0)	4.3 (2.4–6.0)	3 (2.2–5.5)	3.7 (0.8–5.6)	0.541
Urine magnesium (mg/24 h)	82 (58–108)	70 (68–78)	52 (43–70)	97 (77–127)	0.841
Urine oxalate (mg/L)	18.8 (12.6–20.7)	19.9 (14.3–23.5)	5.6 (3.3–17.2)	18.9 (14.8–25.4)	0.490
Urine oxalate (mg/24 h)	28 (24–43)	31 (24–42)	24 (24–40)	41 (32–45)	0.671
Urine citrate (mg/L)	232 (102–344)	255 (116–460)	133 (101–312)	120 (98–328)	0.710
Urine citrate (mg/24 h)	516 (251–790)	501 (215–705)	233 (157–365)	368 (243–956)	0.861
Indwelling time (days)	67 (21–141)	34 (26–40)	55 (22–87)	64 (26–78)	0.043

**Table 6:** Urinary biochemistry (24 h) and indwelling time comparison between patients without encrustations and with calcium oxalate encrustations.

	Encrustation Presence		
	No (n = 25)	Yes—Calcium Oxalate (n = 38)	
Urinary Parameters	Median (IQR)	Median (IQR)	*p*
pH	5.6 (5.2–6.5)	6.00 (5.6–6.7)	0.363
24 h diuresis (mL)	2000 (1450–2700)	1800 (1400–2200)	0.01
Urine creatinine (mg/dL)	57 (38–99)	82 (42–99)	0.093
Urine creatinine (mg/24 h)	1227 (782–1490)	1067 (845–1712)	0.215
Urine urate (mg/dL)	28 (13–43)	34 (18–40)	0.100
Urine urate (mg/24 h)	534 (376–730)	543 (339–749)	0.829
Urine calcium (mg/dL)	6.9 (3.9–10.5)	8.1 (6.6–13)	0.045
Urine calcium (mg/24 h)	132 (89–234)	187 (80–273)	0.399
Urine phosphate (mg/dL)	33 (23–46)	43 (34–68)	0.022
Urine phosphate (mg/24 h)	669 (409–928)	725 (648–937)	0.207
Urine magnesium (mg/dL)	3.4 (2.0–6.2)	3.5 (2.2–6.0)	0.641
Urine magnesium (mg/24 h)	86 (56–117)	82 (58–108)	0.711
Urine oxalate (mg/L)	13.8 (9.6–19.8)	18.8 (12.6–20.7)	0.094
Urine oxalate (mg/24 h)	28 (16–39)	28 (24–43)	0.468
Urine citrate (mg/L)	153 (99–278)	232 (102–344)	0.042
Urine citrate (mg/24 h)	270 (221–608)	516 (251–790)	0.075
Indwelling time (days)	35 (16–92)	67 (21–141)	0.034

**Table 7:** Urinary biochemistry (24 h) and indwelling time comparison between patients without encrustations and with uric acid encrustations.

	Encrustation’s Presence		
	No (n = 25)	Yes—Uric Acid (n = 10)	
Urinary Parameters	Median (IQR)	Median (IQR)	*p*
pH	5.6 (5.2–6.5)	5.3 (5.0–6.4)	0.760
24 h diuresis (mL)	2000 (1450–2700)	1600 (1000–2600)	0.047
Urine creatinine (mg/dL)	57 (38–99)	89 (52–111)	0.230
Urine creatinine (mg/24 h)	1227 (782–1490)	1323 (829–1510)	0.483
Urine urate (mg/dL)	28 (13–43)	50 (29–420)	0.037
Urine urate (mg/24 h)	534 (376–730)	733 (529–3434)	0.167
Urine calcium (mg/dL)	6.9 (3.9–10.5)	6.4 (3.6–12.6)	0.647
Urine calcium (mg/24 h)	132 (89–234)	142 (77–212)	0.184
Urine phosphate (mg/dL)	33 (23–46)	50 (32–75)	0.252
Urine phosphate (mg/24 h)	669 (409–928)	855 (657–1133)	0.492
Urine magnesium (mg/dL)	3.4 (2.0–6.2)	4.3 (2.4–6.0)	0.879
Urine magnesium (mg/24 h)	86 (56–117)	70 (68–78)	0.867
Urine oxalate (mg/L)	13.8 (9.6–19.8)	19.9 (14.3–23.5)	0.297
Urine oxalate (mg/24 h)	28 (16–39)	31 (24–42)	0.741
Urine citrate (mg/L)	153 (99–278)	255 (116–460)	0.859
Urine citrate (mg/24 h)	270 (221–608)	501 (215–705)	0.859
Indwelling time (days)	35 (16–92)	34 (26–40)	0.048

**Table 8:** Urinary biochemistry (24 h) and indwelling time comparison between patients without encrustations and with infectious phosphate encrustations.

	Encrustation Presence		
	No (n = 25)	Yes—Infectious Phosphates (n = 8)	
Urinary Parameters	Median (IQR)	Median (IQR)	*p*
pH	5.6 (5.2–6.5)	6.6 (6.2–6.8)	0.043
24 h diuresis (mL)	2000 (1450–2700)	1533 (1200–1830)	0.807
Urine creatinine (mg/dL)	57 (38–99)	67 (39–90)	0.312
Urine creatinine (mg/24 h)	1227 (782–1490)	1045 (847–1623)	0.224
Urine urate (mg/dL)	28 (13–43)	28 (17–34)	0.443
Urine urate (mg/24 h)	534 (376–730)	417 (298–577)	0.075
Urine calcium (mg/dL)	6.9 (3.9–10.5)	10.5 (6.8–13.7)	0.250
Urine calcium (mg/24 h)	132 (89–234)	167 (78–253)	0.201
Urine phosphate (mg/dL)	33 (23–46)	36 (28–62)	0.697
Urine phosphate (mg/24 h)	669 (409–928)	554 (345–737)	0.683
Urine magnesium (mg/dL)	3.4 (2.0–6.2)	3 (2.2–5.5)	0.876
Urine magnesium (mg/24 h)	86 (56–117)	52 (43–70)	0.554
Urine oxalate (mg/L)	13.8 (9.6–19.8)	5.6 (3.3–17.2)	0.107
Urine oxalate (mg/24 h)	28 (16–39)	24 (24–40)	0.745
Urine citrate (mg/L)	153 (99–278)	133 (101–312)	0.846
Urine citrate (mg/24 h)	270 (221–608)	233 (157–365)	0.344
Indwelling time (days)	35 (16–92)	55 (22–87)	0.068

**Table 9:** Urinary biochemistry (24 h) and indwelling time comparison between patients without encrustations and with non-infectious phosphate encrustations.

	Encrustation Presence		
	No (n = 25)	Yes–Non-Infectious Phosphates (n = 9)	
Urinary Parameters	Median (IQR)	Median (IQR)	*p*
pH	5.6 (5.2–6.5)	5.9 (5.5–7.2)	0.048
24 h diuresis (mL)	2000 (1450–2700)	2050 (1525–2650)	0.807
Urine creatinine (mg/dL)	57 (38–99)	58 (40–64)	0.312
Urine creatinine (mg/24 h)	1227 (782–1490)	1176 (919–1472)	0.224
Urine urate (mg/dL)	28 (13–43)	23 (17–31)	0.443
Urine urate (mg/24 h)	534 (376–730)	448 (414–692)	0.075
Urine calcium (mg/dL)	6.9 (3.9–10.5)	8.5 (7.2–11.1)	0.250
Urine calcium (mg/24 h)	132 (89–234)	227 (131–277)	0.201
Urine phosphate (mg/dL)	33 (23–46)	30 (26–39)	0.697
Urine phosphate (mg/24 h)	669 (409–928)	773 (488–893)	0.683
Urine magnesium (mg/dL)	3.4 (2.0–6.2)	3.7 (0.8–5.6)	0.876
Urine magnesium (mg/24 h)	86 (56–117)	97 (77–127)	0.554
Urine oxalate (mg/L)	14 (10–20)	19 (15–25)	0.317
Urine oxalate (mg/24 h)	28 (16–39)	41 (32–45)	0.453
Urine citrate (mg/L)	153 (99–278)	120 (98–328)	0.846
Urine citrate (mg/24 h)	270 (221–608)	368 (243–956)	0.344
Indwelling time (days)	35 (16–92)	64 (26–78)	0.068

## Data Availability

The data associated with this paper are not publicly available but are available from the corresponding author on reasonable request.
